# Detection of Abnormal Item Based on Time Intervals for Recommender Systems

**DOI:** 10.1155/2014/845897

**Published:** 2014-02-12

**Authors:** Min Gao, Quan Yuan, Bin Ling, Qingyu Xiong

**Affiliations:** ^1^School of Software Engineering, Chongqing University, Chongqing 400044, China; ^2^Key Laboratory of Dependable Service Computing in Cyber Physical Society, Ministry of Education, Chongqing 400044, China; ^3^School of Engineering, University of Portsmouth, Portsmouth PO1 3AH, UK

## Abstract

With the rapid development of e-business, personalized recommendation has become core competence for enterprises to gain profits and improve customer satisfaction. Although collaborative filtering is the most successful approach for building a recommender system, it suffers from “shilling” attacks. In recent years, the research on shilling attacks has been greatly improved. However, the approaches suffer from serious problem in attack model dependency and high computational cost. To solve the problem, an approach for the detection of abnormal item is proposed in this paper. In the paper, two common features of all attack models are analyzed at first. A revised bottom-up discretized approach is then proposed based on time intervals and the features for the detection. The distributions of ratings in different time intervals are compared to detect anomaly based on the calculation of *chi* square distribution (*χ* ^2^). We evaluated our approach on four types of items which are defined according to the life cycles of these items. The experimental results show that the proposed approach achieves a high detection rate with low computational cost when the number of attack profiles is more than 15. It improves the efficiency in shilling attacks detection by narrowing down the suspicious users.

## 1. Introduction

Nowadays, recommender systems have become an effective way to deal with information overload [1]. These systems rely on external ratings and are fully exposed under shilling attacks. Shilling attacks refer to the huge amount of false information input by offenders to affect the system recommendation. Shilling attacks are divided into two types, push attacks and nuke attacks; according to their purposes, the target items are easier or harder to be recommended.

There have been a lot of research on the detection of shilling attacks. The key point of the detection approaches is accuracy and universal applicability. The prior is about detection rate and false alarm rate, and the latter is about the correlation with attack models. The supervised detection algorithms [2–5] are mainly proposed for particular attack models. These methods are not general to all kinds of attack models. Although unsupervised detection algorithms require fewer preconditions and are more general [6], they suffer from high computational cost. To solve these problems, we proposed an approach based on time intervals to detect abnormal items, which is unlimited to any attack models with low computational cost.

To maximize their profits, attackers who make shilling attacks (through injecting fake user profiles) try to increase or decrease the average ratings for targeted items in a short period (the fake users are called shillers or spam users). Therefore, any attack model has two common features: (1) the average ratings of the targeted items will increase or decrease dramatically, and (2) there will be lots of abnormal attack profiles in a short period. Therefore, shilling attacks are detected by not the traditional way, directly finding spam users, but by recognizing abnormal items in our approach. To achieve model-free and effective detection, we then propose an approach based on these features and rating distributions in different time intervals.

The remainder sections of the paper are organized as follows.  Section 2 presents the mostly used shilling attack models and detection algorithms.  Section 3 proposes our model-free approach and four types of items according to their lifecycles.  Section 4 presents the evaluation on dataset MovieLens. Finally, Section 5 draws conclusions.

## 2. Related Works

### 2.1. Shilling Attack Models

There are four mostly used attack models [1, 7, 8] in recommender systems: average attack, random attack, bandwagon attack, and segment attack models.  Table 1 shows the features of the attack models. *I* ^*T*^ is one or a set of target items. For those items, the ratings are *r*_max⁡_ under push attacks and *r*_min⁡_ under nuke attacks.*I* ^*S*^ is a set of selected items that have some relationships with the target items. The ratings for *I* ^*S*^ are *r*_max⁡_ for both push attacks and nuke attacks.*I* ^*F*^ is a set of randomly selected filler items. Under random or bandwagon attacks, the ratings for *I* ^*F*^ are random ratings; under average attacks, the ratings are distributed the mean of each item; under segment attacks, the ratings are *r*_max⁡_ or *r*_min⁡_ for push or nuke attacks, respectively.

### 2.2. Detection Approaches for Shilling Attacks

To detect shilling attacks, several algorithms have been proposed. The earliest detection algorithm was proposed by Chirita et al. [5], in which two attributes were used to recognize attack profiles, namely, RDMA (Rating Deviation from Mean Agreement) and DegSim (Degree of Similarity with Top Neighbors). This algorithm can detect attacks well in a large scale. Mobasher et al. [2] improved RDMA to WDA (Weighted Degree of Agreement) and applied FMTD (Filler Mean Target Difference) metrics for attribute detection. They solved the problem of low detectability for small-sized segment attacks. Afterwards, Williams et al. [4] proposed a series of attributes for intended model and LengthVar (Length Variance) to detect average attacks. These detection approaches are useful to particular attack models; however, attackers can use none of the attack models or hybrid attack models to escape detection.

The above methods are all supervised algorithms, in which shilling attacks are inaccurately detected through classifier. The reason is that these algorithms cope with users individually and do not take the characteristics of the attack group into consideration. Compared with supervised detection algorithms, unsupervised ones, such as PLSA (Probabilistic Latent Semantic Analysis) [9] and VarSelect-PCA (Variable-Selection using Principal Component Analysis) [6], are more applicable to the real conditions of recommender systems because of less conditions and more generalization. These approaches perform the supervised algorithm, but they still suffer from huge computational cost.

Zhang et al. [10] proposed a time series-based approach for shilling attack detection in recommender systems. They detected abnormal items through sample entropy and sample average in rating time sequence, and their experimental results show that the approach has high detection rate and low false alarm rate for the items with dense ratings. The items with sparse ratings are vulnerable to attacks, however, in recommender systems.

From the typical attack models, we find that shilling attacks have two common features: (1) the ratings for the targeted items are either highest or lowest; (2) attack profiles are injected in a relatively short period. Therefore, the average rating of a target item will have dramatic change in a short period. It will be an effective way to find abnormal items through the analysis of time sequences of the items. This approach is attack model-free with low computational cost.

## 3. The Detection Approach Based on Time Intervals

### 3.1. Methodology

Attacks will affect the rating distribution of targeted items in one or several time intervals, and make high ratings or low ratings more intensive no matter what attack models are adopted.

**Definition 1**

An item profile refers to all the ratings on an item order by time. Item profile is a similar concept to user profile.

**Definition 2**

LC (life cycle) [11] refers to the time span from the start of rating time *S* to the final rating time *E* of an item. The value of a life cycle is the timestamp of *E* − *S*.

**Definition 3**

TI (time interval) refers to a period of LC. LC can be divided into *N* intervals in terms of time window *T*. Namely, LC = {*S*, *S* + *T*} + {*S* + *T*, *S* + 2 × *T*} + ⋯+{*S* + (*N* − 1) × *T*, *E*} · {*S* + (*i* − 1) × *T*, *S* + *i* × *T*} (*i* < *N*) is *i*th time interval (TI_*i*_).

**Definition 4**

*DT*_*j*_ refers to the distribution of ratings in the *j*th time interval TI_*j*_ of an item. *DT*_*j*_ = *d*(*r*_*j*_(*i*)); *r*(*i*) is the ratings of item *i*; *r*_*t*_(*i*) is the ratings of item *i* in time interval *t*.

**Definition 5**

*Dw* *T*_*j*_ refers to the distribution of all ratings without TI_*j*_. *DwT*_*j*_ = $d(r_{\overset{-}{t}}(i))$, where $r_{\overset{-}{t}}(i)$ is the ratings of item *i* not in time interval *t*.

The idea of our approach is to compare *DT*_*i*_ with *DwT*_*j*_. If for an item, the *DT*_*i*_ deviates too much from its corresponding *DwT*_*j*_, it will be recognized as an abnormal *DT*_*i*_, and the item will be recognized as an abnormal item. To calculate the similarity between *DT*_*i*_ and *DwT*_*j*_, we revise a bottom-up discretization method [12] based upon chi square distribution *χ* ^2^ test. *χ* ^2^ test is a widely used hypothesis test for enumeration data. It compares between two and more than two sample rates (constituent ratios) and analyzes the correlation of two classified variables. Belonging to nonparametric tests, it decreases the reliance of algorithms upon prior inputs, for the value of *α* can be set according to confidence levels.

The procedures of the proposed approach are as follows.

**Step 1**

Sort ratings for a certain item according to rating time (ascending sort).

**Step 2**

According to time window *t*, the ratings are divided into *n* time intervals.

**Step 3**

Calculate the values of *χ* ^2^ of *DT*_*i*_ and *DwT*_*j*_.

**Step 4**

Set the boundary value for the item according to significant level.

**Step 5**

An interval beyond the boundary value will be marked as a suspicious interval.

The computational formula for *χ* ^2^ is as follows:

(1) χ2=∑i=1m ∑j=1k(Aij−Eij)2Eij,

where *m* is equal to 2 because the comparison is between two distributions: *DT*_*i*_ and *DwT*_*j*_, *k* refers to the quantity of relative classes, *A*_*ij*_ refers to the quantity of the *j*th relative class in TI_*i*_, *E*_*ij*_ refers to *R*_*i*_ × *C*_*j*_/*N*, *R*_*i*_ refers to the total number of all classes in TI_*i*_, *C*_*j*_ refers to the total number of the *j*th relative class in the two intervals. *N* refers to the total number of all *C*_*j*_. For instance, for the ratings from MovieLens dataset, the number of relative class is 5 (*k* = 5) and the degree of freedom *n* is 4.

According to *χ* ^2^ distribution, the significant levels and related boundary values are given in Table 2. For a randomly selected item from MovieLens dataset, given the time interval of the ratings is one month, the monthly rating distributions are shown in Table 3.

As can be seen from Table 2, if the significant level is 0.05, the boundary value will be 9.488. This means any value of *χ* ^2^ exceeding the boundary value is 95% likely to be attacked intervals. In this case, the second month in Table 3 is identified as an attacked interval because its *χ* ^2^ value is 19.501, which is beyond the boundary value 9.488. So, the item is an abnormal item.

### 3.2. The Approach to Select the Size of Time Interval

There are only two preconditions (significant levels and interval size) essential to our detection approach. The number of ratings will increase as the size of time interval increases. If time interval size increases, it will make the rating distributions of all intervals gradually similar to those of the rest ratings. In this case, false alarm rate will decrease gradually, and detection rate will decrease as well, because the algorithm fails to detect small-sized attacks due to an increasing number of ratings. On the contrary, if the time interval size decreases, false alarm rate will increase, and detection rate will increase accordingly.

Consider

(2) χDVj=∑i=1N(m(i,j)−μ(j))2N.

In Formula (2) for *χ* ^2^ deviation value, (*χDV*) is used to find the optimal size of item intervals, where *i* is the *i*th item; *t*_*j*_ is the *j*th size of time interval; *N* = |*I*|; *I* is the set of items.

Consider

(3) m(i,j)=∑k=1nχ2(DTk,DwTk)n=∑k=1nχ2(d(rk(i)),d(rk−(i)))n, n=life  cycle⁡(i)tj,χ2(t,t−)=∑j=1k(At,j−Et,j)2Et,j+∑j=1k(At−,j−Et−,j)2Et−,j,μ(j)=∑i=1Nm(i,j)N.

The purpose of this formula is to find the optimal size of time interval to minimize the average value of the deviation. This size will make the minimum average deviation from every item's *χ* ^2^ value to all items' *χ* ^2^ value. Therefore, the *j*th size of time interval will be selected if *χDV*_*j*_ is the lowest of *χDV*s.

### 3.3. The Classification of Items

To better elaborate the effectiveness of our approach on various items, we divide the items into four categories. For the division, we adopt the concept of product life cycle in marketing. Items can be categorized into four types in accordance with their ratings and life cycles.

**Figure 1:**
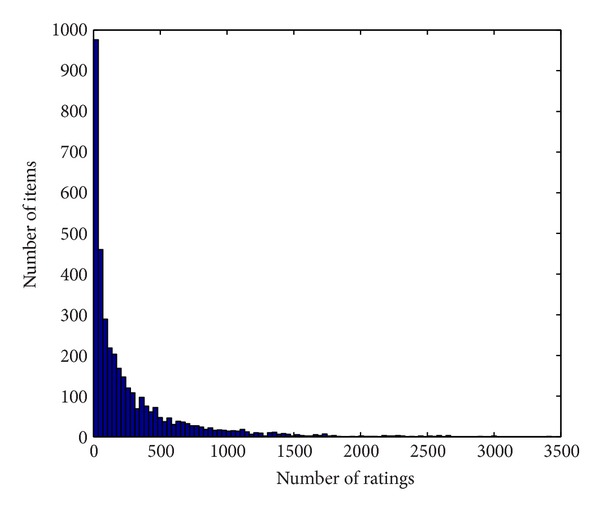
The relationship between the number of ratings and the number of items.

**Figure 2:**
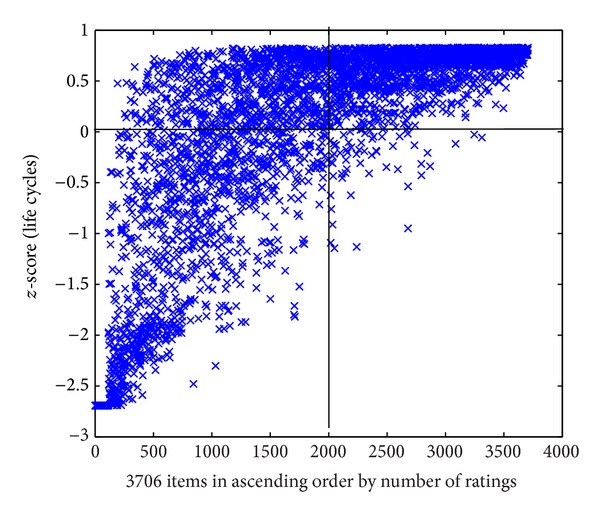
The relationship between the number of ratings and life cycles.

For example, in MovieLens 10 k dataset, there are 1682 movies with 943 users' 100,000 ratings from September 19th, 1997, to April 22nd, 1998. In the dataset, each user has more than 20 ratings, with the range of 1 to 5. The relationships between the numbers of ratings and the numbers of items are shown in Figure 1. As can be seen from Figure 1, the numbers of ratings for the items are noticeably different, and most of them are less than 100.  Figure 2 presents the life cycles of the items in ascending order by the number of ratings. To better compare the items, we normalized the life cycle of all items to be calculated by *z*-score (see formula (4)).

Consider

(4) z-score(x)=x−A−σA,

where *x* is a value of life cycle; $\overset{-}{A}$ is a set of all life cycles; *σ*_*A*_ and *A* are the average value and the standard deviation of *A*, respectively.

As can be seen from Figure 2, items with a long life cycle have a great deal of ratings, while those with a short life cycle were rated less. The items are divided into four parts by *z*-Score (life cycle) (*z*-score = 0) and the average ratings (1000), respectively. According to product type from marketing, the four types are named fad, fashion, style, and scallop, respectively. The items in the left down part belong to fad type. They are popular emerging products. Nevertheless, the numbers of their ratings increase and decline very soon, mainly because they meet customers' temporary requirements rather than long-term needs. The items in the right down part belong to fashion type. A product in this part satisfies few customers, at first. Then the number of ratings grows and declines slowly because it will be gradually accepted by more customers but customers will be attracted by other similar products. The items in the left up part belong to style type. The style items are basic but typical products and will last for a very long time. The items in right up part belong to scallop type. Their life cycles stretch constantly.

## 4. Experimental Evaluation

### 4.1. Dataset

The dataset used in the experiments is widely used Movielens 10 k datasets for recommender systems. The dataset consists of 100,000 ratings (1–5 marks) from 943 users on 1,682 movies. Each user has rated at least 20 movies. To evaluate our approach, for a target item, a certain amount of ratings were injected into the dataset.

We evaluated our approach on the detection rate and false alarm rate [10]. Detection rate refers to the detected attacking events divided by the number of the total attacking events (see (5)). Here, a detected attacking event means that an abnormal interval is detected by the approach. In (5), false alarms mean that normal intervals are recognized as abnormal intervals. False alarm rate is the number of false alarms divided by all alarms (see (6)). In the experiments, we found the most suitable time intervals for those items at first. To better elaborate the effectiveness of our approach, based on the methods mentioned above, we then classified the 1682 items in dataset MovieLens into four categories, which include 524 fad items, 20 fashion items, 622 style items, and 515 scallop items. Finally, we applied our approaches on these four types' items:

(5) Detection  Rate=Number  Of  Detected  Attack  EventsNumber  Of  Total  Attack  Events,

(6) False  Alarm  Rate=Number  Of  False  AlarmsNumber  Of  Normal  Intervals.

### 4.2. The Selection of the Time Interval Size

To find a reasonable size of time interval, we applied the size of time intervals from 5 to 50 and calculated the standard deviation of the average values of *χ* ^2^ in all time intervals for all items, respectively. The steps for finding a reasonable size of time interval are as follows.

**Step 1**

Set *j* = 1 and *t*_*j*_ = 5, where *t*_1_ is the initial size of time interval.

**Step 2**

Consider *n* = lifecycle(*i*)/*t*_*j*_, where *n* is the final time interval.

**Step 3**

Calculate *χ* ^2^ deviation value *χDV*_*j*_.

**Step 4**

Consider *j* = *j* + 1 and *t*_*j*_ = *t*_*j*_ + 5.

**Step 5**

Repeat Step 2 to Step 4 until *j* > 50.

**Step 6**

Find which *j* to minimize *χDV*, then set *t*_*j*_ to be the size of time interval.

**Figure 3:**
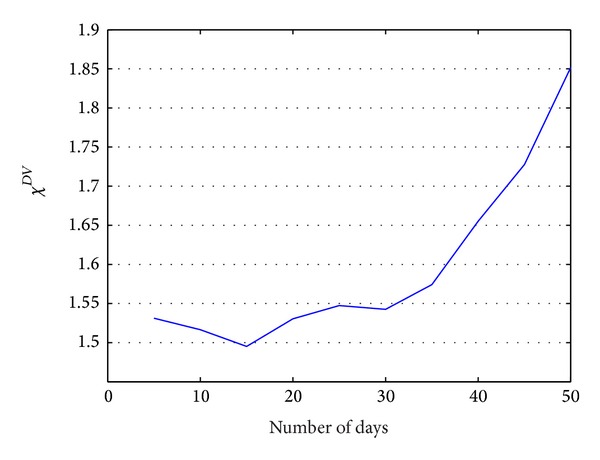
*χ* ^2^ deviation values under different time interval sizes.

As shown in Figure 3, when the size of time interval is 15 days, *χDV* get to be the lowest. The size of time interval was therefore set to 15 days in the our experiments.

### 4.3. Detection of Four Types, Items

As mentioned in Section 2, shilling attacks have two common features: (1) the ratings for the targeted items are either highest or lowest; (2) attack profiles are inserted in a relatively short period. Therefore, the following experiments are based on an assumption that attack profiles are inserted in a time interval. Because our approach is concerned only about the ratings of the each item under detection, the kinds of attack models and filler sizes for attack profiles will not influence the experimental results. In the experiments, all injected ratings are 5 (for push attacks) or 1 (for nuke attacks), which were only injected to target items. Attack sizes were from 5 to 50, that means that from 5 to 50 ratings were injected to the dataset randomly in a same time interval. For every type of items and every attack size, we attacked 50 times. Finally, we got the average detection rates and average false alarm rates of the approach on those four types of items.

**Figure 4:**
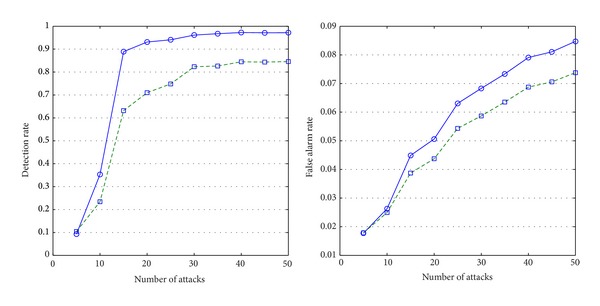
Detection rates and false alarm rates of the fad items.

**Figure 5:**
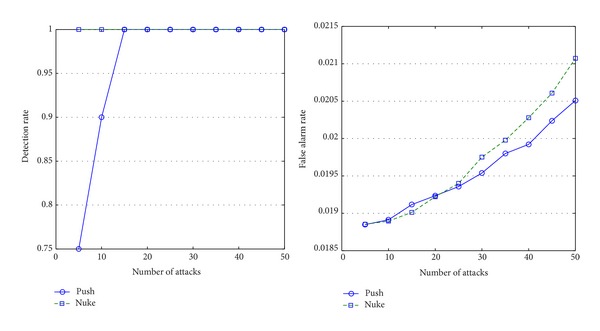
Detection rates and false alarm rates of fashion items.

**Figure 6:**
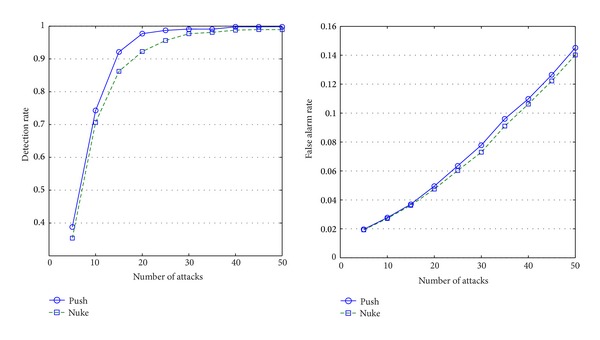
Detection rates and false alarm rates of style items.

**Figure 7:**
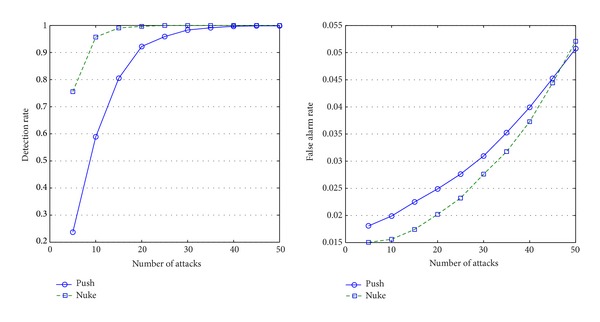
Detection rates and false alarm rates of scallop items.

As can be seen in Figures 4, 5, 6, and 7, the experimental results on these four types show our approach is suitable to different types' items and the detailed analysis is as follows.

1. If attack size is more than 15, the algorithm performs well (the detection rate is around 80%). Please note that, although the algorithm cannot detect abnormal items well on small-size attacks (attack size is less than 10), it is not a serious problem because the small-size attacks cannot influence recommendation lists.
2. As the attack size grows, false alarm rate increases because the ratings' distribution may has been already influenced by the attacks.
3. Items of different types present different features under attacks.
  - Figure 4 shows that push attacks are obviously more detectable than nuke attacks on the items of fad type. That is because the life cycles of those items are last briefly, and most users do not rate them very high.
  - The items of the scallop type are totally different from the items of fad type (see Figure 7). As popular items, their customers increase constantly, and most users will give them high marks. Therefore, nuke attacks are obviously more detectable than push attacks on the item of scallop type.
  - As can be seen in Figure 5, nuke attacks on the items of fashion type are easier detected than the items of other types; this type's items have less false alarms than other types' items. The reason is that the lifecycle of the items of fashion type is short, and their ratings are usually high.
  - The ratings on the items of style type usually lie on two extremes of 1 to 5 marks because customers have totally different opinions on the items. This phenomenon makes the items of style type much easily suffer from attacks than other types' items, and they will have more false alarms than other types' items (see Figure 6).

Additionally, the approach helps to narrow down the suspicious users, because only the users who have rated the item in abnormal intervals will be taken into consideration in the detection of shilling attackers. Thus, if the approach is incorporated into typical detection algorithms, it will decrease their computational cost.

### 4.4. Comparison with Other Approaches

Compared to the approaches proposed by Mobasher et al. [2, 4, 5], our approach has no correlation with attack models and needs low computational cost. Those approaches are only suitable to a particular attack model, but our approach is attack model-free. That is because the attack models and their approaches are all based on user profiles, especially the attack profiles; however, our approach is based only on item profiles.

Compared to the detection approaches based on SVD or PCA [6, 9], our approach needs low computational costs. Supposing that the number of sample vectors is *n* and the number of vector spaces is *d*, the order of time complexity of covariance matrix-based approaches is *O*(*d* ^2∗*n*^), the order of time complexity of PCA-based approaches are *O*(*d* ^3^ + *d* ^2∗*n*^), and our approach's is *O*(*d*∗*n*). Our approach is the lowest because for an item profile, it only uses the profile of the item and need not compare to other items' profiles when analyzing if it is an abnormal item.

Compared to the method of Zhang et al. [10], our approach is more general than their approach. Their method is applicable to the items with dense ratings and long life cycles, but our method is applicable to both the items with dense ratings and the items with sparse ratings. They selected the items (618 in total) with at least 500 ratings from MovieLens 1 M dataset which consists of 1 million ratings. But for most items in recommender systems have sparse ratings and short life cycles. Therefore, our approach is more general than their approach.

## 5. Conclusions

In the paper, we have analyzed two common features of all attack models and defined four types of items. Based on the features and time intervals, a detection approach on abnormal item has been proposed. In the approach, an abnormal item will be recognized if the difference is enough between the distributions between ratings in a time interval and the rest ratings of the item. Because the approach is concern only the ratings of the target item, instead of user profiles, it is free from attack models and filling sizes with low computational cost. The experimental results show that the approach is suitable for all those four types' items. The approach will further narrow down the set of suspicious users and decrease the computational cost of the detection of spam users.

## Figures and Tables

**Table 1:** The features of the attack models.

Attack model	*I* ^*S*^ (selected items)	*I* ^*F*^ (filler items)	*I* ^*T*^ (target items)
Random attack	Ø	*r*(*i* ^*F*^) = random ratings	*r*(*i* ^*T*^) = *r*_max⁡_/*r*_min⁡_
Average attack	Ø	The ratings for *i* ^*F*^ distributed the mean of each item	*r*(*i* ^*T*^) = *r*_max⁡_/*r*_min⁡_
Bandwagon attack	Widely popular items, *r*(*i* ^*S*^) = *r*_max⁡_	*r*(*i* ^*F*^) = random ratings	*r*(*i* ^*T*^) = *r*_max⁡_/*r*_min⁡_
Segment attack	Similar items to target items, *r*(*i* ^*S*^) = *r*_max⁡_	*r*(*i* ^*F*^) = *r*_max⁡_/*r*_min⁡_	*r*(*i* ^*T*^) = *r*_max⁡_/*r*_min⁡_

**Table 2:** Significant levels and related boundary values.

Significant level	0.25	0.10	0.05	0.025	0.01	0.005
Boundary value	5.385	7.779	9.488	11.143	13.277	14.860

**Table 3:** Monthly rating distributions.

*i*th month	Number of rating 1	Number of rating 2	Number of rating 3	Number of rating 4	Number of rating 5	*χ* ^2^
1	0	0	8	3	2	6.4189
2	0	6	9	14	20	19.501
3	1	3	6	6	5	0.7521
4	2	2	9	11	1	7.8567
5	0	1	4	8	0	7.5359
6	1	2	3	1	2	4.3157
7	1	1	8	4	2	3.4811
